# Student Commentary: Targeting the Right Supports to Reduce Pediatric Procedural Pain and Distress

**DOI:** 10.1093/jpepsy/jsac069

**Published:** 2022-09-05

**Authors:** Rachel Fitzpatrick, Brian E McGuire, Helena K Lydon

**Affiliations:** School of Psychology and Centre for Pain Research and Applied Behaviour Research Clinic, National University of Ireland, Ireland; School of Psychology and Centre for Pain Research and Applied Behaviour Research Clinic, National University of Ireland, Ireland; School of Psychology and Centre for Pain Research and Applied Behaviour Research Clinic, National University of Ireland, Ireland

Children often depend on their parents for help and coregulatory support to manage painful and stressful situations, as the capacity for emotion regulation develops gradually across childhood (Noel et al., 2018; Palermo, 2014). In the context of research on needle procedures, a strong body of literature has demonstrated that children’s experience of distress is bidirectionally related to their parent’s behaviors and responses to the needle procedure (Caes et al., 2014; Campbell et al., 2017). In this issue of *JPP*, Constantin and colleagues focused on parent behaviors described as “distress-promoting behaviours” that have been associated with increased child pain during medical procedures (i.e. providing reassurance, empathy, apologizing, and giving control to the child).

One established method of examining the role of emotional regulation in coping with acute pain has been to look at baseline heart rate variability (HRV), whereby low variability is thought to reflect a lack of autonomic flexibility, and this has been associated with higher reports of pain and distress in adults (and vice versa—higher HRV variability is associated with better pain tolerance and reduced distress). In the first pediatric study to explore the role of HRV and acute pain response, Constantin et al. (this issue) examined the associations between child HRV, parent behaviors (reassurance, giving control, and empathy) and child pain, fear, and distress in children aged between 7 and 12 years undergoing venipuncture. As predicted, they found that children with lower HRV experienced greater distress and may be more vulnerable to distress when faced with parental distress-promoting verbalizations during venipuncture. The findings provide a significant advancement in knowledge in pediatric pain and highlight the importance of considering the interplay of biological factors and the social context in shaping a child’s experience of a painful event. However, the findings also raise some methodological, theoretical, and clinical questions.

In terms of methodology, parental behaviors were observed and coded by the research team, including supportive (coping-promoting) behaviors but they were not reported in this article. The integration of these findings within this study would have provided cross-validation if the effect of distress-promoting and distress-reducing parental behaviors were associated with lower and higher HRV, respectively. Of note, a companion publication (Constantin et al., 2022) focused on *parental* HRV and found that parents with good psychological adaptability (high HRV) had *lower* rates of *both* coping-promoting and distress-promoting behaviors (the authors concluded that the parents were not stressed by the procedure). This suggests that parental coping style is an important determinant in the coping supports offered to their children. The clinical status of the children may also be important—62% were described as having a chronic illness, but no further information was provided. Some children may have been more accustomed to venipuncture than others depending on their medical history. The authors also noted that the fact the participants were video recorded may have influenced their responses—this may indeed be a factor, as researcher presence can increase the distress associated with painful events (O’Sullivan et al., 2020). A curious finding also emerged in the study, whereby the physiological responses (HRV) were not closely correlated with the child self-reported levels of pain and distress. The authors identified possible methodological (such as retrospective recall) and theoretical explanations (the two-system framework) for this, but it does highlight an important limitation of the study.

In terms of theoretical aspects, the findings raise questions about why parents choose to utilize certain behaviors with their children. A recent review (O’Sullivan et al., 2021) has highlighted the reciprocal influence of children and caregivers on the other in terms of how children learn to respond to pain in everyday circumstances and such learning will inevitably carry over to clinical settings too. Presumably, parents do not deliberately behave in a way that will increase their child’s distress but have adopted a range of responses based on their child’s personality, emotional maturity, and so on. It may be that parental responses in some settings are more (or less) helpful than in other settings and this warrants further study. It may also be the case that different approaches are required for children who are anxious in general (trait anxiety) versus those who may be anxious purely because of the situation they are in (state anxiety). It is important to note that factors including parental gender or family culture may influence why parents choose to utilize certain behaviors with their children. Socioeconomic factors such as the availability of parental support and the broader social milieu may also influence parental behaviors.

In terms of some of the clinical implications, Constantin et al.’s (2023) findings raise some important questions. For example, how can clinicians address the biological factors and the social context in order to improve children’s experiences of medical procedures? One possible explanation for the development of fear/distress or pain associated with a needle procedure in children is that of classical conditioning—children can quickly learn to associate an unpleasant experience (e.g., pain) with a particular context (e.g., hospital). It is possible that clinicians can advise parents on simple changes to parent–child communications that may reduce fear and distress for their child and generate more positive associations. Parental verbal behaviors which focus on the procedure can increase distress for children. Focusing the child’s attention on something other than the procedure, by engaging in nonprocedural talk, humor, or other distraction techniques may be helpful. Distraction techniques can range from watching cartoons, playing video games, hospital clowns, distraction cards, reading a book, and listening to music, which are all effective techniques in reducing pain and anxiety (e.g. Inan & Inal, 2019; Kuo et al., 2018; Míguez-Navarro & Guerrero-Márquez, 2016; Newell et al., 2018). In recent years, virtual reality has been found to be more effective than usual treatments (e.g. physical/verbal comfort) or other distraction techniques (e.g. kaleidoscope) at reducing pain and anxiety during venipuncture in children (Atzori et al., 2018; Chan et al., 2019; Özalp Gerçeker et al., 2020). In order to address the physiological response associated with emotional regulation as measured by HRV, relaxation training may be beneficial for children who present with low HRV. There may also be opportunities to transfer strategies across settings, for example, a recent Delphi study (Wallwork et al., 2022) identified a range of caregiver responses that experts considered important in helping children to cope with everyday pain and injury, and some of these techniques may be helpful either in the venipuncture setting itself, or in the broader context of teaching children to cope with pain, including procedural pain.

The important study by Constantin et al. (2023) has identified several potential avenues for research and clinical practice that may be helpful in reducing pain and distress in children during venipuncture. The findings from this study demonstrate the right supports are needed to ensure a decrease in procedural distress. Future research should focus on skill development in the area of relaxation techniques which may be beneficial to children who present with low HRV as these techniques may result in higher HRV resulting in less procedural distress for pediatric patients.

*Conflicts of interest*: None declared.
